# Organizational aspects of selective analysers

**DOI:** 10.1155/S1463924688000392

**Published:** 1988

**Authors:** T. D. Geary

**Affiliations:** Institute of Medical and Veterinary Science Adelaide South Australia Australia


Journal of Automatic Chemistry Vol. 10, No. 4 (October–December 1988), pp. 184–187

### Organizational aspects of selective analysers

**T. D. Geary**
*Institute of Medical and Veterinary Science, Adelaide, South Australia, Australia*

It was intended that this symposium should provide information to assist laboratory staff to understand a few of the characteristics of analytical systems. This paper will concentrate on the importance of sample identification and bi-directional interfacing, the relative merits of selectivity against batch selectivity, a review of the quality-control needs of the newer analysers, and, finally, the paper will address the advantages and disadvantages of open and closed systems. These characteristics should be considered when selecting analytical instruments for the laboratory.

The selection process adopted by the laboratory prior to purchase requires a careful review of the characteristics of the intruments under consideration and a clear definition of the problem the laboratory wishes to solve by the purchase. There are several publications in the literature which discuss procedures to follow in order to define the laboratory's problems and then to find an analyser most suited to help solve the problems so defined. The Expert Panel on Instrumentation has published papers from a symposium [1] on the topic and a brief review of the procedure suggested would not be out of place in the context of the present publication. A decision strategy was outlined by Professor Buttner in the previous symposium and it is intended here to concentrate on two of the points made: the definition of the problem and the interaction with the infrastructure.

Professor Buttner's discussed non-monetary criteria to be used in the selection process and provided a listing of points relating to the practicability of the instrument. These points are listed below:

(1) Space requirements.
(2) Energy consumption, other service requirements.
(3) Ease of operation.
(4) Adaptability and change-over of analytical methods.
(5) Possibility of introducing urgent samples into routine procedure.
(6) No restriction in terms of the selection of reagents.
(7) Safe operation.
(8) Fault detection and signalling.
(9) Full operating instructions.
(10) Environmental aspects, for example production of effluent.

An understanding of the topics discussed in that presentation will assist with the collection of data on the important points, such as ease of operation, adaptability of methods, handling of the urgent specimen, open or closed systems, fault detection and signalling the fault.

The interaction with the infrastructure is a difficult subject for it is often hard to predict the effect that any alteration in the laboratory organization will have on the handling of the workload, particularly when one considers the wide ranging changes positive identification, bi-directional interfacing, selectivity and the computing power of modern analytical systems do have on the operation of a laboratory.

When analysing the total effect the purchase of a new system will have, importance should be placed upon positive identification. The advantages provided by positive identification of all steps from specimen collection to sample presentation to the analyser should be appreciated. The International Federation of Clinical Chemistry and the European Committee for Clinical Laboratory Standards have a Working Party with a mandate to consider specimen identification. This is chaired by Professor Bonini and a draft document produced by the Working Party contains details of the various approaches taken to positive identification. However, attempts to provide a total hospital positive patient identification scheme have not been particularly successful. This is unfortunate for the potential value of such a system is great. The manufacturers have tended to concentrate upon systems for positive identification which begin when the specimen reaches the laboratory. These systems provide the laboratory with a number of advantages but even they cannot be fully implemented without the availability of bi-directional interfacing (figure 1). The communication between computers presents something of a problem which can only be overcome by further standardization of the communication protocol.

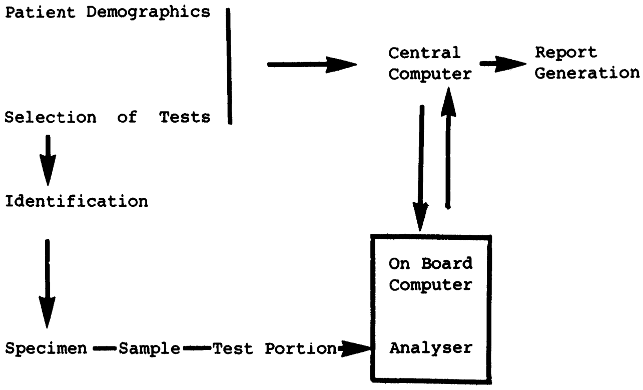

*Figure 1.*

The flow diagrams of both selective analysers and selective batch analysis are shown in figures 2 and 3. As these diagrams indicate there are advantages in the use of the direct selective analysis against the selective batch analysis. These advantages include the following:

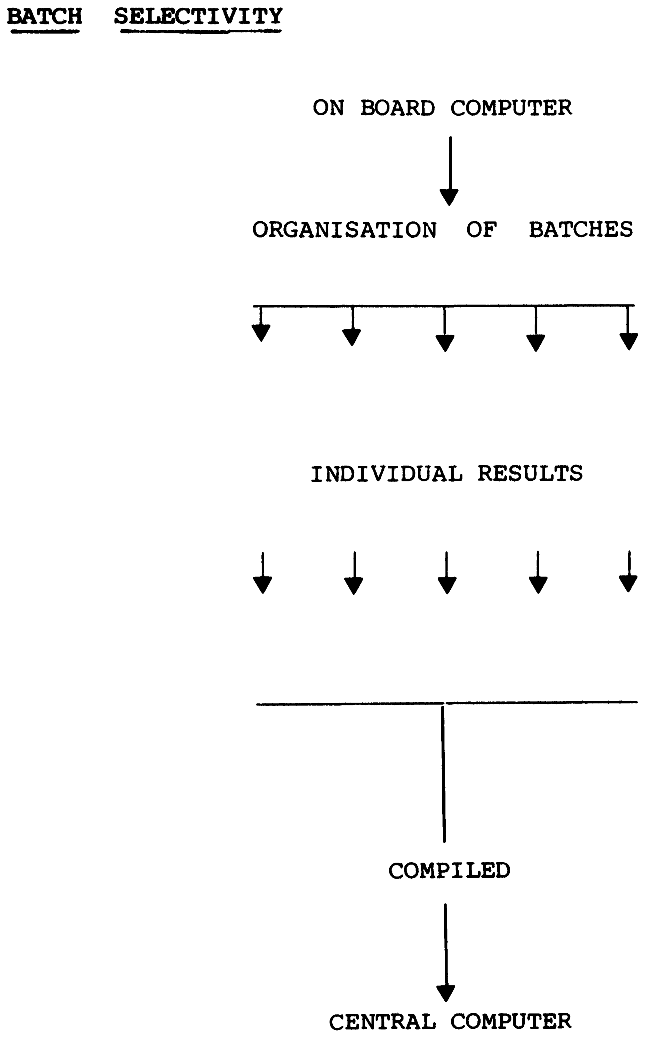

*Figure 2.*

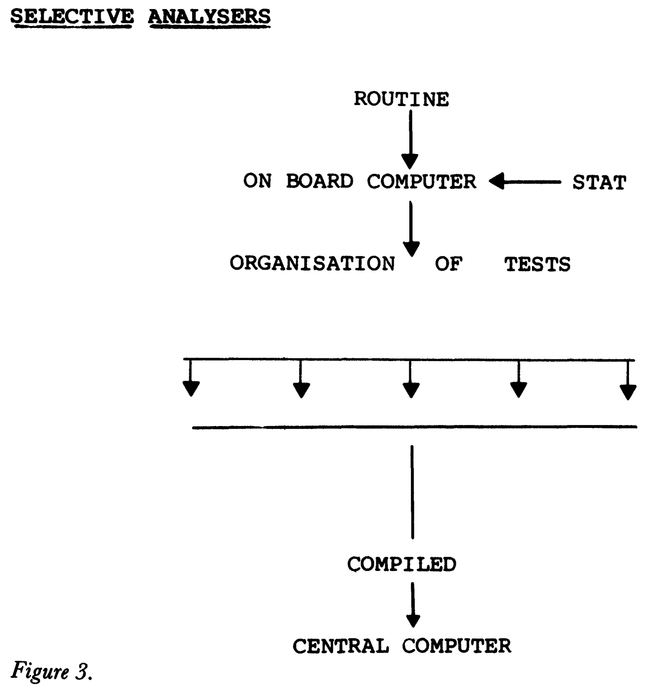

*Figure 3.*

(1) A reduction in the dwell time of each patient's results because in the selective analysis the tests requested for each patient are completed before the tests on the next sample are started.
(2) New specimens can be added at any stage in the process without interfering with the work flow on the analyser.
(3) Stat specimens similarly can be added at any time and processing of the tests begins immediately after the completion of the tests on the previous specimen.
(4) Bi-directional interfacing provides, in the selective mode, a facility which would allow for the recall of previous results for each patient with immediate viewing and analysis of any analytical changes which may have occurred.

There are disadvantages, such as the possibility of reagent carry-over and reduced sample rate.

The next point to be discussed is internal quality control. There is a need to review the whole concept of internal control. This is nowhere more obvious than in the situation of the selective analyser. If the traditional approach to quality control is used, a very large number of quality control specimens would have to be run. There has been an evolution in the use of quality control in the clinical laboratory (figure 4), first with the acceptance of

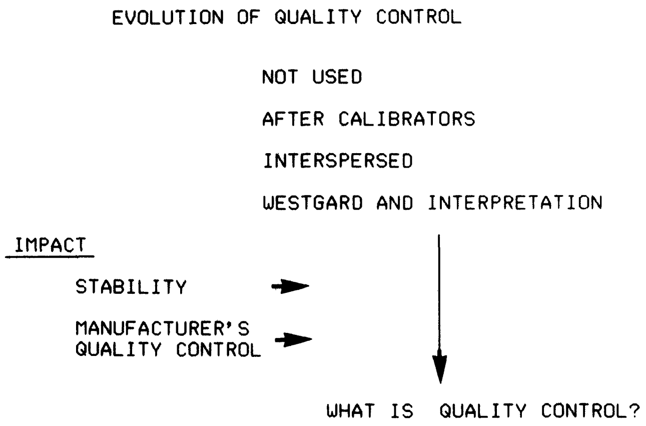

*Figure 4.*

the need, then the developments which led to more effective program design and techniques, following which there was better understanding and interpretation of the results. However, the role played by quality control must be reconsidered in the light of advances such as the stability inherent in the new analytical systems and the commitment to quality of manufacture by the industry. The Black Box allows the laboratory staff to present a sample and receive an answer. If one considers the following points, the question can be asked, why are the laboratories using quality-control materials to check the performance of closed analytical systems?

(1) Quality-control materials are used to check the performance of the analytical systems.
(2) The quality control material does not give a true indication of the pre-analytical errors because it is a control known to the operator and so consciously or subconsciously may be treated more carefully.

(3) The systems are largely operator independent, allowing a sample to be presented and an analytical result is produced.
(4) Some systems recalibrate every time a sample is presented; and others have calibration which is stable for three or more months.
(5) The manufacturer applies a very stringent quality control programme 'in house'.
(6) The manufacturers have accepted the responsibility for the quality of the analytical system.
(7) The analytical performance of the systems as shown by the use of quality-control material is acceptable.
(8) For some analytes quality-control materials have been shown to react differently to the patient sample.

The above question should be put because there is a tendency among the staff of laboratories to adopt a traditional attitude to internal quality control, which does not reflect the advances made in analytical techniques.

The manufacturer is in a position to display a considerable amount of information with respect to the performance of various components of the analytical system. These are available through the service modes. Taking this fact into account and coupling it with the extensive effort which is put into guaranteeing the quality of the reagent systems in use, one wonders if it would not be possible to devise a quality assurance system for the laboratory which is independent of the conventional use of quality control material. The on-board services checks could be monitored and a warning displayed if there was a change in performance of clinical significance.

The question of how analytical rejection criteria are established is the next point for discussion. In our laboratory a Kodak EK 700 is monitored for its performance and the analytical performance data for the quality-control material are used to determine the rejection criteria by calculating the mean and standard deviation. Using this method of determining the criteria to be used for acceptance or rejection of analytical batches leads to the figures in table 1. If these are compared to the performance criteria in the literature they are narrow. Some examples of performance criteria are given also in table 1. The 10%, 20% and 50% refers to the performance of the laboratories in those percentile in the Australian Chemical Pathology Quality Assurance Programme. The data listed under 'best' refer to the most stringent performance criteria among published work. The 'goal' refers to performance criteria taking into account intra- and inter-biological variance. The principle of establishing acceptable limits of analytical performance by calculating the mean and standard deviation from the existing performance of the system should be questioned. As can be seen from table 1, the performance of the Kodak EK 700 system is tight for some analytes and rejection based upon those criteria may not be sensible. There is a need to consider the clinical requirements. In addition, the frequency of use of quality control materials should be reviewed. If one was to look at an analyser which has been available for a number of years, the Technicon SMAC, the quality of performance as determined by the quality control programmes on the system at the Institute of Medical and Veterinary Science is good and the results would indicate that perhaps there is a need to review the internal quality control programme. Specimens are inserted in the following sequence; recalibration step, two levels of quality control material, 16 specimens, two levels of quality control material, 8 specimens, two levels of quality control material, recalibration step. As mentioned above, the quality control data indicate that less stringent control would be warranted. As a general statement, the principles of quality control are not reviewed often enough to ensure that the procedures for monitoring the quality of the analytical data are keeping abreast of advances in analytical techniques. The point to be made is that with the introduction of new techniques and new analytical systems the existing laboratory operations should be reviewed to ensure that they are not restricting the appropriate utilization of the system.

The final point to be covered in this section of the symposium's papers relates to the advantages and disadvantages of open and closed analytical systems operating in the selective analysis mode. From the points listed in tables 2 and 3, it becomes clear that a decision

*Table 1. Imprecision data for a carrier bound analytical system CV as percentage.*

| Analyte | 700 | 10% | 20% | 50% | Best | Goal |
|---|---|---|---|---|---|---|
| Sodium | 0·79 | 0·5 | 0·6 | 0·9 | 0·9 | 0·4 |
| Pot. | 1·26 | 0·8 | 1·0 | 1·6 | 1·5 | 2·2 |
| Ca | 1·24 | 0·9 | 1·3 | 2·1 | 1·45 | 0·9 |
| Chlor. | 1·07 | 0·8 | 1·0 | 1·4 | 1·0 | 1·1 |
| Gluc. | 1·05 | 1·4 | 1·9 | 3·0 | 2·7 | 2·2 |

*Table 2. Open analytical systems.*

ADVANTAGES
- Greater flexibility
- Wider range of reagents
- Research applications
- Often consumables cost less
- Range of tests more extensive

DISADVANTAGES
- More dependent on operator's skills
- Manufacturer's QC not available
- Requires skills to troubleshoot

*Table 3. Closed analytical system.*

ADVANTAGES
- Manufacturer Quality Assurance
- Tendency for operator independence
- Positive identification of reagents

DISADVANTAGES
- Routine analytical tool
- Often consumables cost more
- Reliance on Manufacturer's QA
- Reliance on Manufacturers investigations
- The 'Black Box'
- Range of tests often restricted has to be made with respect to the role that the analytical system is to play in the laboratory because open systems have advantages if operated in a research or developmental mode. The inherent flexibility of analysers which operate in the selective mode should be weighed against the possible decrease in the sample rate of the system if it is operated in this mode.

In conclusion, it is important that positive identification, bi-directional interfacing, selectivity and the value of resident chemistries be understood, as these are facilities which are integral to the effectiveness of the laboratory organization and should be considered carefully in the purchase of analytical systems. In addition, there is a need to review the design and operation of internal quality control programmes in light of the performance characteristics of the systems available to the laboratory.

### Summary

Selective analysers have a significant impact on the organization of the laboratory and there are areas where the systems will continue to develop and extend the range of facilities available.

In this respect, positive identification, bi-directional interfacing and on-board interpretative programmes will play a major role in extending the value to the laboratory. The stability of the analytical systems has led to a questioning of the more traditional approach to quality control.

When seeking to obtain the optimum value from an analytical system there is a need to affirm how quality control is to be applied and to make greater use of the facilities available in diagnostic and the service modes of these systems. The self-monitoring checks available in the various operating modes should be considered as a form of quality control.

Selective analysers need to be understood for the potential of these systems to be fully utilized.

### Reference

1. *Journal of Automatic Chemistry*, **2**, 1 (1980).

**NEW PUBLICATION**

AUTOMATIC METHODS OF ANALYSIS

*By M. Valcárcel and M. D. Luque de Castro (xii + 560 pages, US$ 131·50/Dfl. 250·00, ISBN 0-444-43005-9).*

This new monograph published in August 1988 provides a comprehensive overview of the state of the art of the automation of laboratory processes in analytical chemistry. The topics have been chosen according to such criteria as the degree of consolidation, scope of application and most promising trends.

The first part of the book begins with the basic principles behind the automation of laboratory processes, then describes automatic systems for sampling and sample treatment. In the second part the principal types of analysers are discussed: continuous, batch and robotic. The third part is devoted to the automation of analytical instrumentation: spectroscopic, electroanalytical and chromatographic techniques and titrators. The last part presents some examples of the application of automation to clinical chemistry, environmental pollution monitoring and industrial process control.

*Details from booksellers or Elsevier Science Publishers, PO Box 211, 1000 AE Amsterdam, The Netherlands.*

**P S ANALYTICAL EXTENDS ITS RANGE OF AUTOSAMPLES**

As part of its ongoing development programme P S Analytical has just introduced a new product in its autosampler range. Aimed at the Atomic Absorption market the new PSA 20.030 Autosampler has a sample bottle capacity of 27 ml. The containers used are constructed from polypropylene and have a re-usable seal, making them ideal for sample preparation and other alternate analyses, prior to use on the sampler. This new unit is based on the proven PSA 20.020 design utilizing the patented wash pot capability. Every position on the tray can then be used for a standard or sample solution, increasing the throughput of samples.

The PSA 20.030 Autosampler is fully compatible with the complete P S Analytical product range. It can therefore be interfaced into a fully automated fluorescence system and controlled by the 'Touchstone' software package. A simple TTL-logic interface enables the equipment to link to other instrument makers equipment and computer systems. It is an ideal product for OEM applications.

*For further details contact John Bangerter* at P S Analytical Ltd, Arthur House, Cray Avenue, Orpington, Kent BR5 3TR, UK.

